# Glycolysis and Oxidative Phosphorylation Play Critical Roles in Natural Killer Cell Receptor-Mediated Natural Killer Cell Functions

**DOI:** 10.3389/fimmu.2020.00202

**Published:** 2020-02-20

**Authors:** Zixi Wang, Di Guan, Shu Wang, Louis Yi Ann Chai, Shengli Xu, Kong-Peng Lam

**Affiliations:** ^1^Department of Microbiology and Immunology, Yong Loo Lin School of Medicine, National University of Singapore, Singapore, Singapore; ^2^Bioprocessing Technology Institute, Agency for Science, Technology and Research, Singapore, Singapore; ^3^NUS Graduate School for Integrative Sciences & Engineering (NGS), National University of Singapore, Singapore, Singapore; ^4^Department of Biological Sciences, National University of Singapore, Singapore, Singapore; ^5^Division of Infectious Diseases, University Medicine Cluster, National University Health System, Singapore, Singapore; ^6^Department of Medicine, Yong Loo Lin School of Medicine, National University of Singapore, Singapore, Singapore; ^7^Department of Physiology, Yong Loo Lin School of Medicine, National University of Singapore, Singapore, Singapore

**Keywords:** natural killer cells, anti-tumor, cell metabolism, glycolysis, CD16, NKG2D

## Abstract

Natural killer (NK) cells are innate lymphocytes that directly kill tumor and pathogen-infected cells upon activation by cytokines and NK cell receptors (NKRs) without previous sensitization. It is known that cell metabolism affects the differentiation and effector functions of immune cells. For instance, interleukin−2 and interleukin−15 treatment increases glycolysis and oxidative phosphorylation (OXPHOS) in NK cells to support their effector functions. However, little is known about the metabolic reprogramming of human NK cells upon their activation by NKRs. In this study, we investigated the metabolism of NK cells stimulated via NKRs. We found that NK cells upregulated glycolysis and OXPHOS in response to anti-CD16 antibody or NKG2D ligand engagement. Inhibition of either glycolysis or OXPHOS impaired NK cell production of interferon-γ. Interestingly, inhibition of glycolysis but not OXPHOS decreased NK cell killing and dampened NK cell degranulation and Fas ligand expression, suggesting that glycolysis is more critical for NKR-activated cell cytotoxicity. Thus, our study provides insight into understanding the metabolic requirements underlying different effector functions of human NK cells.

## Introduction

Natural killer (NK) cells are innate lymphocytes that comprise 5–15% of human peripheral blood mononuclear cells (PBMCs) (1). They are considered as the first line of immune defense because they can destroy tumor and virus-infected cells without priming (2). Resting NK cells patrol the blood and can also infiltrate into tissues to exert effector functions when activated (3). NK cells kill target cells by releasing lytic granules that contain granzymes and perforins as well as activating the death receptors on target cells (4, 5). They also secrete chemokines and cytokines, such as interferon-γ (IFN-γ) and tumor necrosis factor-α (TNF-α), to shape both innate and adaptive anti-virus or anti-tumor immune responses (6).

NK cell functions are regulated by cytokines [e.g., interleukin (IL)-2, IL-15, and IL-18] and NK cell receptors (NKRs). NKRs can be divided into two broad categories, namely activating NKRs and inhibitory NKRs, based on the signals that they transduce. Activating NKRs (e.g., NKG2D, NKp30, and CD226) recognize specific activating ligands to transduce activating signals. On the other hand, inhibitory NKRs (e.g., NKG2A and KIR2DL) mainly detect major histocompatibility complex (MHC) class I molecules on potential target cells and transduce inhibitory signals to antagonize the activating signals (7). Whether NK cells become activated or not depends on the balance of activating and inhibitory signals. Healthy host cells express MHC class I molecules but low levels of activating ligands, thus delivering overall stronger inhibitory signals to prevent NK cells from being activated. However, unhealthy cells, including tumor and virus-infected cells, upregulate stress-induced activating ligands, or down-regulate their MHC class I molecules to escape from cytotoxic T cell killing. Hence, the activating signals override the inhibitory signals received by NK cells, leading to NK cell activation and the elimination of unhealthy cells (8). Among the activating NKRs, CD16 induction of antibody-dependent cellular cytotoxicity and NKG2D-mediated recognition of target cells are two important modes of tumor elimination by NK cells (9).

Activated immune cells have high demands for ATP molecules for energy consumption and nutrients for anabolic synthesis to cater for their effector functions (10). Glycolysis and oxidative phosphorylation (OXPHOS) are two major metabolic pathways to provide energy for cells. Glycolysis converts glucose into pyruvate via a series of metabolic reactions. This process is oxygen-independent, and it is a relatively inefficient way of ATP generation compared with OXPHOS. However, glycolysis is found to be the dominant metabolic pathway in pro-inflammatory cells, possibly because it could be rapidly activated via the induction of glycolytic enzymes and could provide intermediates for cell biosynthesis (10). Recent studies have demonstrated that resting NK cells depend mainly on OXPHOS for their survival (11, 12). When activated by cytokines, such as IL-2, IL-12, and IL-15, both murine and human NK cells upregulate glycolysis and OXPHOS to support IFN-γ production (12, 13). In contrast, inhibitory cytokine TGF-β, which is commonly found in the tumor microenvironment, is shown to suppress NK cell metabolism and effector functions (14).

However, the metabolic requirements for NKR-induced human NK cell activation remain mostly unknown. Here, we show that human NK cells display increased glycolysis and OXPHOS upon anti-CD16 antibody and NKG2D ligand stimulation, and inhibition of either glycolysis or OXPHOS dampens NKR-induced IFN-γ secretion. In addition, we found that NKR-induced NK cell cytotoxicity mainly depends on glycolysis that supports NK cell degranulation and Fas ligand (FasL) expression.

## Materials and Methods

### NK Cell Purification and *ex vivo* Expansion

NK cells were obtained from human PBMCs and were expanded as previously described. Briefly, blood samples were obtained from healthy donors with written consent and were approved by the Institutional Review Board of National University of Singapore (08-300). PBMCs were isolated by gradient centrifugation and re-suspended in GMP Serum-free Stem Cell Growth Medium (SCGM, CellGenix) supplemented with 10% fetal bovine serum (FBS, Biowest). K562 cells (ATCC) were genetically modified to express membrane-bound (mb) IL-15, mbIL-21, and 4-1BB ligand (15) and were maintained in IMDM medium (Life Tech) with 10% FBS and γ-irradiated before use. PBMCs and irradiated K562 cells were co-cultured at the ratio of 1:2 in 10 ml of complete medium with human recombinant IL-2 (50 IU/ml) at D0. At day 7, NK cells were re-stimulated by K562 feeder cells at the ratio of 1:1. At day 14, NK cells were selectively expanded to about 1,000-fold and were used for experiments. Freshly isolated primary NK cells were purified from PBMCs by negative selection using EasySep™ human NK cell isolation kit (STEMCELL Technologies) according to the manufacturer's protocol.

### NK Cell Activation

Anti-2B4 (clone C1.7, 3 μg/ml; BioLegend) and anti-CD16 antibody (clone 3G8, 15 μg/ml; BioLegend), as well as NKG2D ligand MICA (R&D system, 2.5 μg/ml) and ULBP1 (R&D system, 2.5 μg/ml), and LFA-1 ligand ICAM-1 (R&D system, 2.5 μg/ml) were used to stimulate NK cells. Antibodies and ligands were diluted with PBS and coated on 6-well and 24-well plates at 4°C overnight. After incubation, plates were washed once with PBS. NK cells were added to the coated plate and incubated at 37°C (5% CO2) for 4 or 6 h as indicated. Cells were harvested for subsequent metabolic and flow cytometry analyses.

### ECAR and OCR Analysis

An XF-24 Extracellular Flux Analyzer (Seahorse Bioscience) was used for real-time analyses of extracellular acidification rate (ECAR) and oxygen consumption rate (OCR) of NK cells according to the manufacturer's protocol. Briefly, NK cells were collected after stimulation and resuspended in XF base and assay medium (Agilent Technologies) for ECAR and OCR analysis, respectively. Cells were adhered to CellTaq (BD Pharmingen) coated XF 96-well microplate (Seahorse Bioscience) at 200,000 cells per well. Cells were starved in a non-CO2 chamber at 37°C for 1 h to deplete all the stored glucose in NK cells. ECAR was measured under basal conditions followed by sequential addition of 10 mM glucose, 0.5 μM oligomycin, and 100 mM 2-deoxyglucose (2-DG). This procedure allows an estimation of extracellular acidification caused by non-glycolytic acidification, glycolysis, and glycolytic capacity of NK cells. OCR was measured under basal conditions followed by the injections of oligomycin (1 μM), FCCP (1 μM), and rotenone (500 nM) plus antimycin (500 nM). This protocol allows the accurate calculation of oxygen consumption due to basal respiration, maximal respiration, ATP production and non-mitochondrial respiration.

### Flow Cytometry

Cells were treated with 2-DG (30 mM), or oligomycin (2.5 μM) plus rotenone (500 nM) and antimycin (500 nM) (Sigma-Aldrich) for 4 h in a humidified incubator at 37°C (5% CO_2_). For glucose-free treatment, NK cells were cultured in glucose-free RPMI-1640 medium (Life Technologies) supplemented with 10% FBS overnight. Subsequently, cells were stimulated with antibodies or ligands as stated above in a 24-well plate at 37°C (5% CO_2_) for 4 h. When indicated, the pretreated NK cells were washed twice with PBS before stimulated with K562 cells at effector to target (E:T) ratio of 1:2 for 30 min. For glucose uptake assay, cells were cultured in glucose-free RPMI 1640 medium supplemented with 10% FBS and 2-NBDG (30 μM, Life Technologies) for 1 h at 37°C (5% CO_2_). Cells were then harvested and stained for 20 min on ice with saturating concentration of antibodies for surface staining. Intracellular staining was performed using cytofix/cytoperm kit (BD Pharmingen) according to the manufacturer's protocol. Antibodies used were as follows: PE/BUV395-CD3, PE-Cy™7/BUV395-CD56, PE-FasL, APC-TRAIL, PE-Cy™7-IFN-γ (BD Biosciences), FITC-Streptavidin, PerCP-CD16, FITC-CD107a (BioLegend), Biotin-NKG2D (eBioscience). Live cells were gated according to their forward scatter (FSC-A) and side scatter (SSC-A), and single cells were selected based on FSC-W and FSC-A. NK cells were identified as CD3^−^CD56^+^ cells.

### Quantitative RT-PCR

One million NK cells were left untreated or stimulated as indicated above in a 24-well plate at 37°C (5% CO_2_) for 2 h. Cells were then collected and washed with ice-cold PBS once. RNA was isolated from cells using TRIzol (Life Technologies, Qiagen) according to the manufacturer's instructions. cDNA was generated with Oligo(dT)18 Primer (Thermo Fisher Scientific). Relative quantification of IFN-γ expression was analyzed by quantitative real-time PCR using SYBR Select Master Mix (Applied Biosystems). GAPDH housekeeping gene was used as the internal standard. Amplification was performed using primers at 300 nM for 40–80 cycles (15 s at 95°C and 30 s at 60°C). The following primers were used:

GAPDH Forward: 5′-GAAGGTGAAGGTCGGAGT -3′,

Reverse 5′-CATGGGTGGAATCATATTGGAA-3′;

IFN-γ Forward 5′-AAAAATAATGCAGAGCCAAATTG-3′,

Reverse 5′-TAGCTGCTGGCGACAGTTCA-3′.

A melting curve was performed at the end to confirm the specificity of the amplification. Each reaction was analyzed in duplicates. Relative expression of IFN-γ mRNA was calculated using ΔΔCt.

### ELISA

Cells were pretreated with metabolic inhibitors and stimulated with antibodies or ligands as indicated above in a 24-well plate at 37°C (5% CO_2_) for 4 or 6 h as indicated. The supernatant was collected and assayed for IFN-γ detection using Human IFN-γ ELISA MAX™ Standard Set (Biolegend, San Diego, CA). Granzyme B secretion was measured using Human Granzyme B ELISA development kit (Mabtech) according to the manufacturer's protocol.

### Cytotoxic Assay

NK cells were pretreated with 2-DG (30 mM), or oligomycin (2.5 μM) plus rotenone (500 nM) and antimycin (500 nM) for 4 h or pre-incubated overnight with glucose-free PRMI-1640 medium at 37°C (5% CO_2_). Cells were then co-cultured with cell tracer violet-labeled K562 cells at E:T ratio of 0.25:1, 0.5:1, and 1:1 in the presence of metabolic inhibitors for 1.5 h at 37°C (5% CO_2_). When indicated, pretreated NK cells were washed with PBS twice before co-culturing with K562 cells in medium without metabolic inhibitors. For K562-pretreated killing assay, K562 cells were treated with metabolic inhibitors stated above for 1 h and washed twice with PBS before co-culturing with untreated NK cells for 1 h at E:T ratio of 0.25:1, 0.5:1, and 1:1. To detect dead cells, cells in the co-culture were collected and labeled with fixable viability dye eFluor 780 (FVD, eBioscience) in PBS for 10 min at room temperature before flow cytometry analysis. The percentage of killing by NK cells was calculated using the following equation:

$$\begin{array}{l} \%\;Killing=\\ \frac{\%Dead\;K562_{\left(E:T\;ratio=n:1\right)}-\;\%Dead\;K562_{\left(E:T\;ratio=0:1\right)}}{100\%-\%Dead\;K562_{\left(E:T\;ratio=0:1\right)}} \end{array}$$

### Statistical Analysis

Statistical analysis was performed with GraphPad Prism 6. Independent sample Student's *t*-test was used to compare the means of 2 groups. One-way and two-way ANOVA tests were used to compare means for more than 2 groups, and Dunnett's tests were used for multiple comparisons. *P*-value < 0.05 was considered as significant. ^*^P < 0.05, ^**^P < 0.01, and ^***^P < 0.001.

## Results

### NK Cells Upregulate Glycolysis and OXPHOS in Response to NKR Stimulation

*Ex vivo* expanded NK cells are widely used in NK-cell based immunotherapy in autologous and allogeneic transfer settings (8). This expansion process is achieved by co-culturing NK cells with engineered feeder cells that express membrane-bound (mb) cytokines and co-stimulatory molecules. After expansion, NK cells are enriched in number and are more potent in eliminating tumor cells (16). In our study, we co-cultured PBMCs with irradiated feeder cells expressing mbIL-15, mbIL-21, and 4-1 BB ligand in medium supplemented with IL-2. After 7 days of culture, NK cell number increased ~70-fold and was further increased to 1,000-fold at day 14 of culture. The purity of CD3^−^CD56^+^ NK cells is more than 90%, and about 70% of these cells express CD16 and NKG2D (Figure S1).

**Figure 1:**
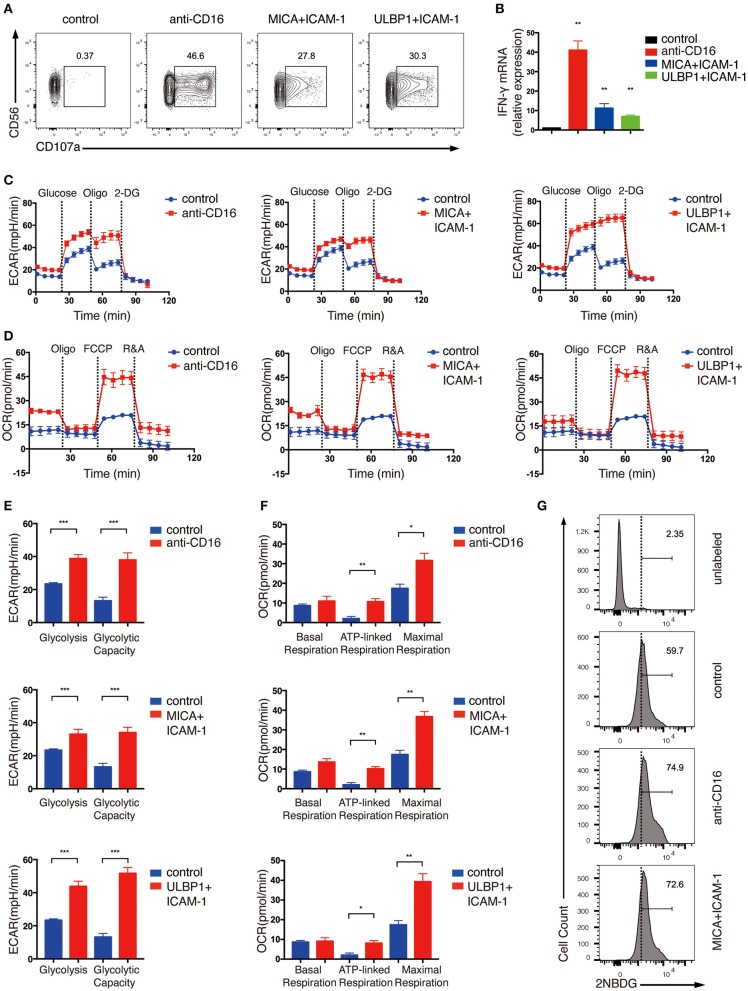
*Ex vivo* expanded NK cells upregulate glycolysis and OXPHOS in response to activating NKR stimulation. *Ex vivo* expanded NK cells were stimulated by anti-CD16 monoclonal antibody (mAb), MICA plus ICAM-1, or ULBP1 plus ICAM-1 for 4 h. NK cell activation was examined by **(A)** surface CD107 a expression as determined by flow cytometry and **(B)** IFN-γ mRNA level as quantified by RT-PCR. **(C,E)** Analyses of extracellular acidification rate (ECAR) in NK cells without (blue) and with (red) stimulation by anti-CD16 mAb, MICA plus ICAM-1, or ULBP1 plus ICAM-1. ECAR of NK cells with sequential additional of glucose (10 mM), the mitochondrial ATP synthase inhibitor oligomycin (oligo) (0.5 μM) and the glucose analog 2-deoxyglucose (2-DG) (100 mM) were assessed in real time **(C)**. The ECAR before addition of glucose or after addition of 2-DG represents the non-glycolytic acidification of NK cells. Glycolysis was quantified as the difference of non-glycolytic acidification and ECAR after addition of glucose; glycolytic capacity is calculated by the change of ECAR before and after addition of 2-DG **(E)**. **(D,F)** Oxygen consumption rate (OCR) of unstimulated and stimulated cells as indicated above. Real-time changes of OCR in NK cells with addition of oligomycin (1 μM), the mitochondrial uncoupler FCCP (1 μM), and inhibitors of mitochondrial electron transport chain complex I and III, rotenone (500 nM) and antimycin (500 nM) (R&A), were measured **(D)**. OCR of NK cells after the addition of R&A represents non-mitochondrial respiration. Basal respiration was quantified as the difference of non-mitochondrial respiration and OCR before addition of oligo. ATP-linked respiration was calculated by the change of OCR before and after addition of oligo. Maximal respiration was estimated by the difference of OCR before and after addition of R&A **(F)**. **(G)** Flow cytometry analysis of glucose intake by NK cells with or without CD16 or NKG2D engagement. Stimulated and unstimulated NK cells were labeled with the fluorescent glucose analog 2-NBDG. The unstimulated cells without 2-NBDG incubation (denoted unlabeled) served as the negative control. Data were presented as Mean ± SEM [*n* = 2 for **(B)** and *n* = 3–6 for **(C–F)**]. Samples were compared using One-way ANOVA with Dunnett's test **(B)** and independent Student's *t*-test **(E,F)**. *P < 0.05, **P < 0.01, ***P < 0.001.

Next, we examined the metabolic changes in the *ex vivo* expanded NK cells upon NKR stimulation. NK cell activation requires the co-stimulation of multiple NKRs except for CD16 which can activate NK cells by itself (17). Thus, we stimulated NK cells with anti-CD16 antibody alone or with NKG2D ligand (ULBP1 or MICA) plus intercellular adhesion molecule-1 (ICAM-1), which induces the adhesion of NK cells to the target and the polarization of cytolytic granules in NK cells. NK cell activation was successfully induced by these treatments as shown by their upregulated expression of the degranulation marker lysosomal-associated membrane protein-1 (CD107a) and IFN-γ mRNA (Figures 1A,B).

To determine the metabolic changes associated with NKR activation of NK cells, we examined extracellular acidification rate (ECAR) and oxygen consumption rate (OCR), which enable direct quantification of glycolysis and mitochondrial respiration, respectively, in NK cells treated with CD16 or NKG2D engagement. As shown in Figures 1C–F, NK cells upregulated both ECAR (Figures 1C,E) and OCR (Figures 1D,F) in response to NKR stimulation. The glycolysis rate of NK cells almost doubled upon CD16 and NKG2D engagement (Figures 1C,E). Consistent with this phenomenon, NKR-stimulated NK cells also uptake more glucose than unstimulated cells (Figure 1G). Glycolytic capacity represents the maximum rate of glycolysis that cells can achieve. It is measured by using oligomycin (oligo), an ATP synthase inhibitor that inhibits ATP production from OXPHOS, to ensure that glycolysis is the only process that supplies ATP for cells. The ECAR in the expanded NK cells was not further increased when the ATP production in OXPHOS was inhibited using oligomycin, suggesting that these cells have mostly met their total ATP needs through glycolysis (Figures 1C,E).

We observed an increase in basal mitochondrial respiration in NK cells stimulated by anti-CD16 and MICA plus ICAM-1 compared with unstimulated cells (Figures 1D,F). The ATP-linked respiration was low in unstimulated *ex vivo* expanded NK cells, which further indicated that these cells relied heavily on glycolysis for ATP production. The NKR-stimulated NK cells, however, were more active in ATP-linked respiration than the unstimulated cells. The maximal respiration can be assessed by the addition of an uncoupler FCCP, which drives the respiratory chain to operate at maximum capacity. NK cells stimulated by anti-CD16 antibody and NKG2D ligand significantly enhanced their maximal respiration, indicating that these NK cells have more spare respiratory capacity to meet higher energy demand upon NKR engagement (Figures 1D,F).

**Figure 2:**
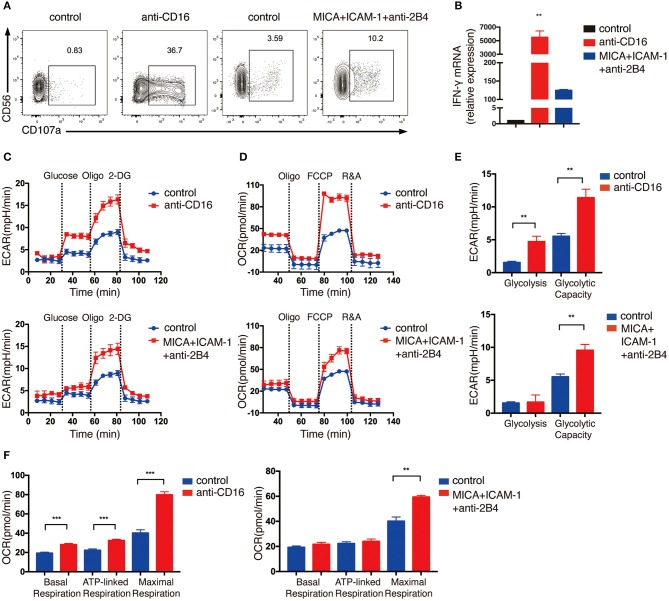
Freshly isolated primary NK cells upregulate glycolysis and OXPHOS in response to activating NKR stimulation. NK cells were isolated from human PBMCs and stimulated by either anti-CD16 mAb or MICA plus ICAM-1 and anti-2B4 mAb for 4 h. NK cell activation was examined by **(A)** surface CD107a expression as determined by flow cytometry and **(B)** IFN-γ mRNA level as quantified by RT-PCR. **(C,D)** ECAR and OCR analyses performed using Seahorse Extracellular Flux Analyzer as described in Figure 1. Real-time changes of ECAR **(C)** and OCR **(D)** in NK cells without (blue) and with (red) stimulation by anti-CD16 mAb (upper panel) or MICA plus ICAM-1 and anti-2B4 (lower panel) were measured. **(E)** Quantification of glycolysis and glycolytic capacity of unstimulated and stimulated NK cells. **(F)** Quantification of basal respiration, ATP-linked respiration and maximal respiration in NK cells with and without NKR stimulation. Data were presented as Mean ± SEM [*n* = 2 for **(B)** and *n* = 3–5 for **(C–F)**]. Samples were compared using One-way ANOVA with Dunnett's multiple comparisons test **(B)** and independent Student's *t*-test **(E,F)**. **P < 0.01, ***P < 0.001.

Because the expansion of NK cells using feeder cells would probably change the metabolic state of NK cells, thus the expanded NK cells may exhibit different metabolic responses from resting primary human NK cells upon NKR simulation. Therefore, we also performed ECAR and OCR analyses on primary NK cells that were freshly isolated from human PBMCs. Because primary NK cells are less active than the expanded NK cells, we stimulated primary NK cells with anti-CD16 antibody alone, or co-engaged three NK cell receptors using MICA, anti-2B4 antibody and ICAM-1 to induce stronger activating signals for optimal cell activation. The NK cells were successfully activated by these treatments, as indicated by their upregulation of CD107a and IFN-γ mRNA expression (Figures 2A,B). Unlike expanded NK cells, the glycolytic rate of freshly isolated primary NK cells was further enhanced when the ATP production in OXPHOS was inhibited by oligomycin, indicating that the primary NK cells depend on both glycolysis and OXPHOS to provide ATP and had relatively lower glycolytic machinery compared with the expanded NK cells (Figure 2C). Similar to the *ex vivo* expanded NK cells, we found freshly isolated NK cells to also exhibit significantly increased glycolysis, glycolytic capacity, and OXPHOS upon NKR stimulation (Figures 2C–F).

Taken together, these data indicate that glycolysis and OXPHOS are both elevated in *ex vivo* expanded and freshly isolated NK cells upon NKR activation.

### NKR-Induced NK Cell Production of IFN-γ Requires Glycolysis and OXPHOS

**Figure 3:**
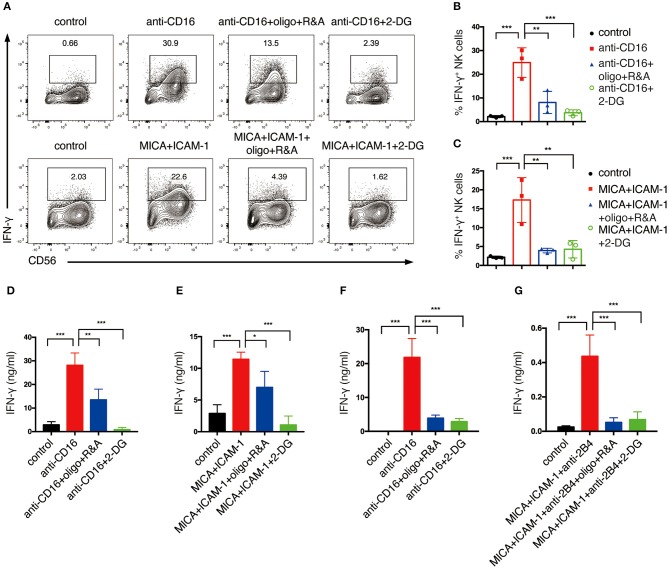
Glycolysis and OXPHOS are required for NKR-induced IFN-γ production of both *ex vivo* expanded and freshly isolated NK cells. *Ex vivo* expanded NK cells were untreated or pretreated with oligomycin plus rotenone and antimycin (oligo+R&A) or 2-DG for 4 h and then stimulated with anti-CD16 mAb or MICA plus ICAM-1 for 4 h. **(A)** Flow cytometry analysis of IFN-γ production of NK cells. **(B,C)** Quantification of the percentage of IFN-γ-expressing NK cells after various stimulations and treatments as indicated. **(D,E)** IFN-γ secretion of expanded NK cells after various stimulations and treatments as indicated. Supernatant were collected after stimulation and IFN-γ was detected by ELISA. **(F,G)** IFN-γ secretion of freshly isolated primary human NK cells. Freshly isolated NK cells were pretreated with oligo+R&A or 2-DG for 4 h and then stimulated with anti-CD16 mAb or MICA plus ICAM-1+anti-2B4 mAb for 6 h. Culture supernatant were collected and IFN-γ was measured by ELISA. Data were presented as Mean ± SD [*n* = 3 for **(B–G)**]. Samples were compared using One-way ANOVA with Dunnett's test. *P < 0.05, **P < 0.01, ***P < 0.001.

We next explored how NKR-induced metabolic changes affect NK cell functions. One of the major effector functions of NK cells is the secretion of cytokines, e.g., IFN-γ. Hence, we examined how cell metabolism affects NK cell production of IFN-γ upon NKR stimulation. We treated NK cells with 2-deoxyglucose (2-DG), which is an analog of glucose that blocks glycolysis. To inhibit OXPHOS, we treated NK cells with mitochondrial ATP synthase inhibitor oligomycin, mitochondrial electron transport chain complex I inhibitor, rotenone, and complex III inhibitor, antimycin (R&A). Anti-CD16 antibody and NKG2D ligand engagement successfully induced IFN-γ production in expanded NK cells as examined by flow cytometry analysis (Figures 3A–C). However, NKR-induced IFN-γ production was significantly reduced by the inhibition of glycolysis and OXPHOS. ELISA measurements of IFN-γ also showed that the inhibition of either OXPHOS or glycolysis decreased IFN-γ secretion of both expanded (Figures 3D,E) and freshly isolated NK cells (Figures 3F,G) upon NKR engagement. These data suggest that both glycolysis and OXPHOS are required for IFN-γ production upon NKR activation of NK cells.

### Glycolysis Supports NK Cell Cytotoxicity

As cytotoxicity is another important effector function of NK cell, we proceeded to explore how cell metabolism affects NK cell cytotoxicity. The chronic myeloid leukemia cell line K562, which lacks MHC class I and expresses abundant activating NKR ligands, is an ideal target for the assessment of NK cell cytotoxicity (18). Due to limiting access to freshly isolated primary human NK cells, we mainly focused on *ex vivo* expanded NK cells in assessing NK cell cytotoxicity. This approach is also consistent with the protocol adapted for cell therapy, i.e., the use of *ex vivo* expanded NK cells for infusion into cancer patients.

**Figure 4:**
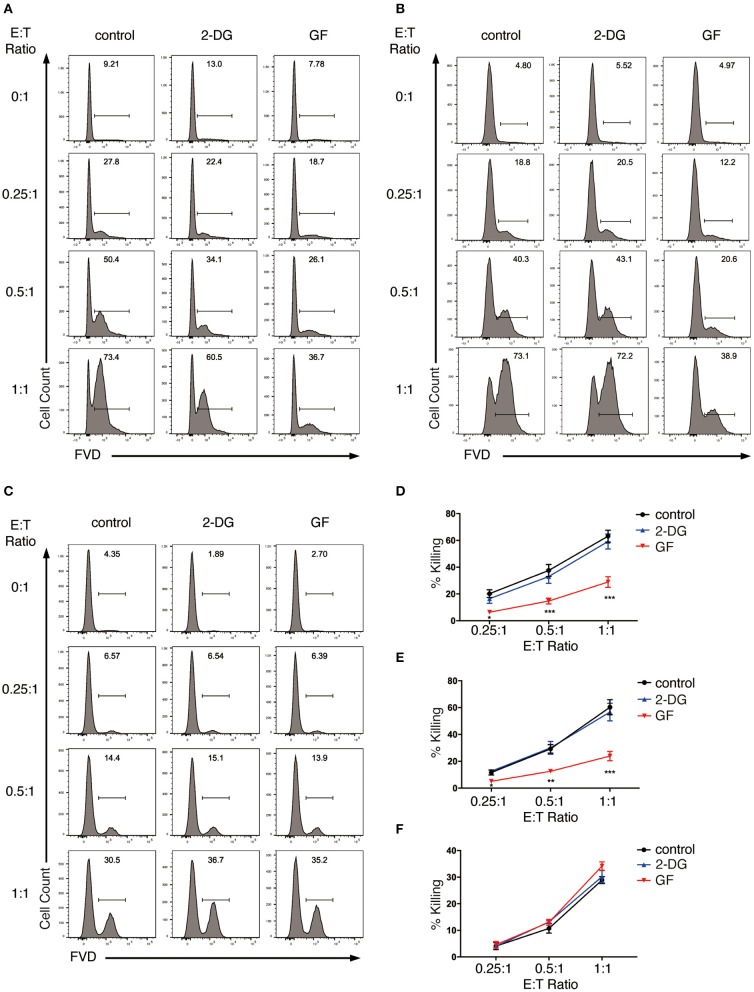
Glycolysis supports NK cell cytotoxicity against target K562 cells. **(A,B)** Killing of K562 cells by glycolysis-inhibited NK cells. *Ex vivo* expanded NK cells were pretreated for 4 h with 2-DG or incubated overnight with glucose-free (GF) medium to inhibit glycolysis. The pretreated NK cells were then **(A)** co-cultured with cell tracer violet-labeled K562 cells in the presence of glycolysis inhibitors (i.e., 2-DG or glucose-free medium), or **(B)** washed twice with PBS before co-culturing with K562 cells in the absence of glycolysis inhibitors for 1.5 h at various effector to target (E:T) ratios. Untreated NK cells co-cultured with K562 cells served as control. Dead K562 cells were labeled with Fixable Viability Dyes (FVD). **(C)** Killing of glycolysis-inhibited K562 cells by NK cells. K562 cells were left untreated or pretreated with 2-DG or glucose-free medium for 1 h to inhibit glycolysis. These K562 cells were then washed twice with PBS before co-culturing with NK cells at various E:T ratios for 1 h. Dead K562 cells were labeled with FVD. **(D–F)** Quantification of the percentage of killing of K562 cells by NK cells as shown in **(A–C)**, respectively. Data were presented as Mean ± SEM of 3–10 independent experiments [*n* = 7 for **(D)**, *n* = 10 for **(E)**, *n* = 3 for **(F)**]. Samples were compared using two-way ANOVA with Dunnett's multiple comparisons test. *P < 0.05, **P < 0.01, ***P < 0.001.

We first analyzed the effect of glycolysis inhibition on NK cell cytotoxicity. We started with the short-term inhibition of glycolysis by treating NK cells with 2-DG for 4 h. These NK cells were then co-cultured with K562 cells in the presence of 2-DG at effector to target (E:T) ratio of 0.25:1, 0.5:1, and 1:1 for the killing assays. NK cells without metabolic inhibition exerted potent cytotoxicity toward K562 cells, as more than 60% of the target cells were killed within 1.5 h of incubation at E:T ratio of 1:1 (Figures 4A,D). 2-DG treatment only showed a slight decrease in the killing of K562 cells, which is not statistically significant, indicating that short-term treatment of 2-DG has little impact on NK cell cytotoxicity. We next introduced a longer-term inhibition of glycolysis by incubating NK cells with glucose-free medium overnight. Inhibiting glycolysis by glucose starvation significantly reduced the killing of K562 cells by NK cells at all E:T ratios examined, with the most drastic decrease observed at E:T ratio of 1:1 (Figures 4A,D). These data indicate that long-term inhibition of glycolysis impaired NK cell cytotoxicity.

In the above studies, the metabolic inhibitors were present in the co-culture, thereby affecting not only effector but also target cells. To examine if applying metabolic inhibitors to NK cells alone affects NK cell cytotoxicity, we washed the pretreated NK cells twice with PBS and co-cultured them with untreated K562 cells in culture medium free of glycolysis inhibitors. Similarly, glucose starvation impaired NK cell cytotoxicity at all E:T ratios examined, with the most drastic decrease at E:T ratio of 1:1 (Figures 4B,E). To investigate if inhibition of glycolysis also affects K562 cells' susceptibility to NK cytotoxicity, we pretreated K562 cells with 2-DG or glucose-free medium for 1 h before co-culturing them with NK cells. Inhibiting glycolysis in K562 cells had no impact on their susceptibility to NK cell cytotoxicity (Figures 4C,F).

Taken together, these data indicate that glycolysis is critical in supporting the cytotoxicity of *ex vivo* expanded NK cells.

### OXPHOS Is Not Required for NK Cell Cytotoxicity but Inhibition of OXPHOS Renders K562 Cells More Susceptible to NK Cell Cytotoxicity

**Figure 5:**
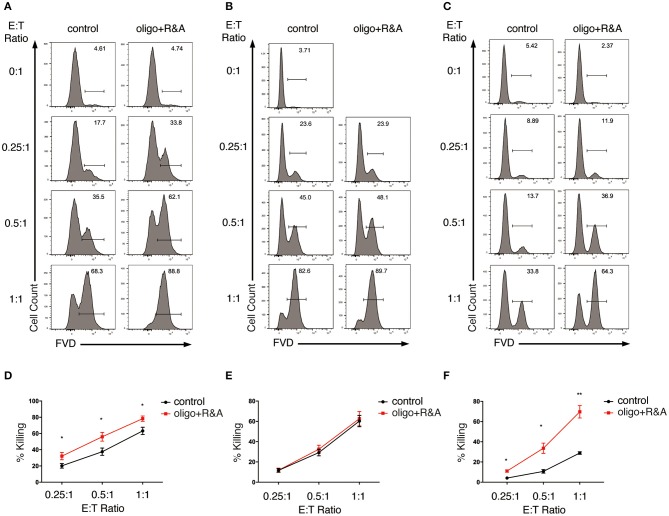
OXPHOS is not required for NK cell cytotoxicity but inhibition of OXPHOS renders K562 cells more susceptible to NK cell cytotoxicity. **(A,B)** Killing of K562 cells by OXPHOS-inhibited NK cells. *Ex vivo* expanded NK cells were pretreated for 4 h with oligo plus R&A to inhibit OXPHOS. The pretreated NK cells were **(A)** co-cultured with cell tracer violet labeled-K562 cells in the presence of oligo plus R&A, or **(B)** washed twice with PBS before co-culturing with K562 cells in the absence of oligo plus R&A for 1.5 h at various E:T ratios. Untreated NK cells co-cultured with K562 cells served as control. Dead K562 cells were labeled with FVD. **(C)** Killing of OXPHOS-inhibited K562 cells by NK cells. K562 cells were left untreated or pretreated for 1 h with oligo plus R&A to inhibit OXPHOS. These K562 cells were washed twice with PBS before co-culturing with NK cells at various E:T ratios for 1 h. **(D–F)** Quantification of the percentage of killing of K562 cells by NK cells as shown in **(A–C)**, respectively. Data were presented as Mean ± SEM of 3–10 independent experiments [*n* = 7 for **(D)**, *n* = 10 for **(E)**, *n* = 3 for **(F)**]. Samples were compared using independent Student's *t*-test. *P < 0.05, **P < 0.01.

Next, we explored the impact of OXPHOS inhibition on NK cell cytotoxicity. We pretreated NK cells with oligo plus R&A for 4 h to inhibit OXPHOS. These NK cells were then co-cultured with K562 cells in the presence of oligo plus R&A at various E:T ratios. Although untreated NK cells were already potent in killing K562 cells, we were surprised to observe a drastic increase in the killing of K562 cells at all E:T ratios examined when OXPHOS was inhibited (Figures 5A,D). OXPHOS-inhibited NK cells exhibited comparable killing of K562 cells at E:T ratio of 0.25:1 and 0.5:1 to that of untreated NK cells at E:T ratio of 0.5:1 and 1:1, respectively. However, when we washed off the metabolic inhibitors before incubating the pretreated NK cells with K562 cells, OXPHOS inhibition showed little effect on NK cell cytotoxicity, as they exhibited similar percentage of killing as untreated NK cells (Figures 5B,E).

Our data above indicated that inhibition of OXPHOS had little impact on NK cell cytotoxicity when applied to NK cells alone but increased the killing of K562 target cells when given in co-culture. Hence, we next examined if the metabolic inhibitors could change target cells' susceptibility to NK cell cytotoxicity. We pretreated K562 cells with oligo plus R&A for 1 h to inhibit OXPHOS. The inhibitors were washed away before co-culturing the pretreated K562 cells with untreated NK cells. Interestingly, inhibiting OXPHOS rendered K562 cells more susceptible to NK cell cytotoxicity (Figures 5C,F). Therefore, inhibiting OXPHOS enhanced the susceptibility of K562 cells to NK cell killing without compromising NK cell cytotoxicity, suggesting that the inhibition of OXPHOS in tumor environment might be advantageous for NK cell-based immunotherapy.

### Glycolysis Supports NKR-Induced NK Cell Degranulation and Granzyme B Production

Granule exocytosis pathway is one of the major pathways mediating NK cell cytotoxicity (19). Activated NK cells and their target cells form immunological synapses, which facilitate the entry of lytic granules that contain perforin and granzymes into the target cells to induce cell apoptosis via caspase-dependent or -independent pathways. This process is called degranulation and is associated with the expression of CD107a on NK cell surface (19).

**Figure 6:**
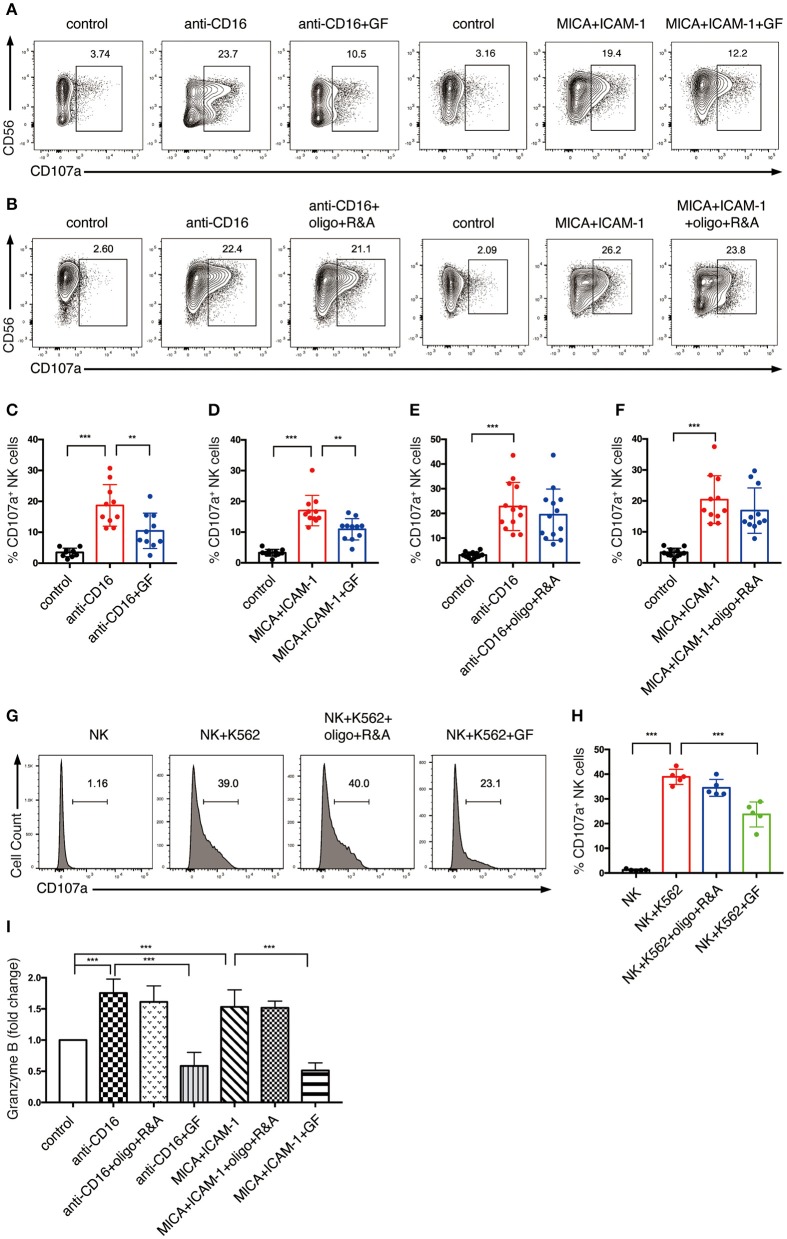
NK cell degranulation induced by NKR activation is dependent on glycolysis. **(A–F)** The effect of glycolysis or OXPHOS inhibition on CD107a expression of NK cells induced by anti-CD16 mAb or MICA plus ICAM-1. *Ex vivo* expanded NK cells were cultured in glucose-free (GF) medium overnight or pretreated with oligo+R&A for 4 h or left untreated. These NK cells were stimulated with anti-CD16 mAb or MICA plus ICAM-1 for 4 h. The CD107a expression of NK cells were examined by flow cytometry **(A,B)**. **(C–F)** Quantification of the percentage of CD107a-expressing NK cells. **(I)** Granzyme B secretion of NK cells subjected to various stimulations and treatments as stated above. Granzyme B secreted by NK cells was quantified using ELISA, and results were normalized to untreated NK cells. **(G,H)** The effect of inhibition of glycolysis or OXPHOS on CD107a expression of NK cells induced by K562 cells. Untreated and the pretreated NK cells were washed twice with PBS and then co-cultured with cell tracer violet-labeled K562 cells for 30 min. CD107a expression of NK cells were examined by flow cytometry **(G)**. **(H)** Quantification of the percentage of CD107a-expressing NK cells induced by K562 cells. Data were presented as Mean ± SD [*n* = 10–13 for **(C–F)**; *n* = 5 for **(H,I)**]. Samples were compared using one-way ANOVA with Dunnett's test. **P < 0.01, ***P < 0.001.

Thus, we explored how cell metabolism affects the granule exocytosis pathway. We pre-cultured NK cells with glucose-free medium overnight or pretreated them with oligo plus R&A for 4 h to inhibit glycolysis and OXPHOS, respectively. Subsequently, we stimulated these cells with anti-CD16 antibody or NKG2D ligand. NKR stimulation induced the upregulation of CD107a expression, which increased from 3% in unstimulated NK cells to around 20% in NKR-stimulated NK cells (Figures 6A–F). The CD107a induction was significantly suppressed by glucose-free treatment, which dropped to around 10% (Figures 6A,C,D). In contrast, OXPHOS-inhibited NK cells did not show a significant decrease in CD107a expression (Figures 6B,E,F). Similarly, the K562 cell-induced expression of CD107a on NK cells was also dramatically reduced by glycolysis inhibition but was not affected by OXPHOS inhibition (Figures 6G,H).

We also investigated the impact of cell metabolism on granzyme B secretion of NK cells. Our ELISA data indicated that anti-CD16 antibody and NKG2D ligand increased the granzyme B secretion of NK cells (Figure 6I). Similar to the results of CD107a expression, granzyme B secretion induced by NKR-activation was abrogated by the inhibition of glycolysis but not OXPHOS. Taken together, our data suggest that glycolysis is essential in supporting NK cell degranulation induced by NKR activation, whereas OXPHOS has little impact on this process.

### Glycolysis Supports NKR-Induced FasL Expression

Death receptor pathways also contribute to NK cell cytotoxicity. NK cells express death receptor ligands—FasL, TNF, and TNF-related apoptosis-inducing ligand (TRAIL), which recognize the corresponding receptors Fas, TNFR, and TRAILR, respectively, on target cells. Upon receptor-ligand interaction, death-inducing signaling complexes are formed to activate caspase-dependent and -independent pathways that eventually induce cell apoptosis (19).

**Figure 7:**
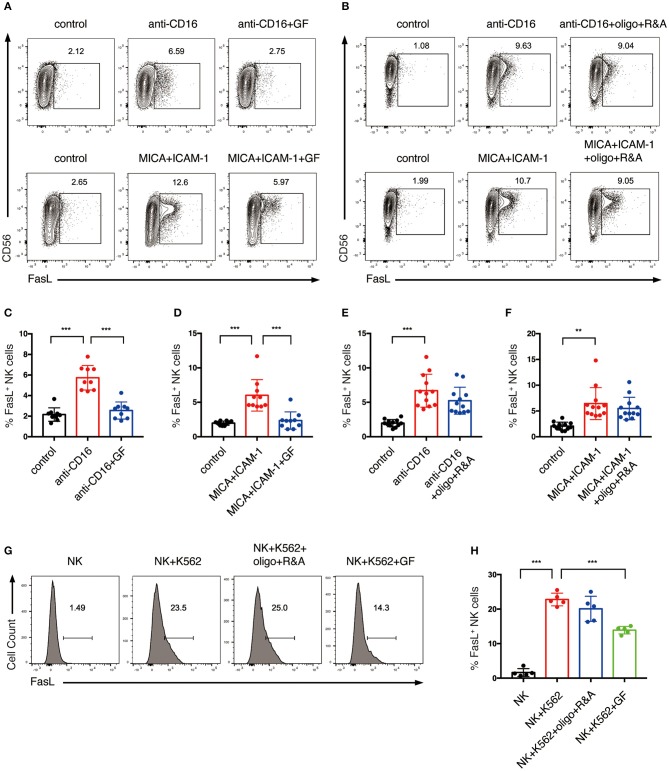
Glycolysis supports FasL expression on NK cells activated by NKR. **(A–F)** The effect of glycolysis or OXPHOS inhibition on FasL expression of NK cells induced by anti-CD16 mAb or MICA plus ICAM-1. Expanded NK cells were cultured in glucose-free medium overnight or pretreated with oligo+R&A for 4 h or left untreated. Untreated and the pretreated NK cells were stimulated with anti-CD16 mAb or MICA plus ICAM-1 for 4 h. FasL expression of NK cells were examined by flow cytometry **(A,B)**. **(C–F)** Quantification of the percentage of FasL-expressing NK cells. **(G,H)** The effect of inhibition of glycolysis or OXPHOS on K562 cell-induced FasL expression of NK cells. NK cells were pretreated to inhibit glycolysis or OXPHOS as stated above. Untreated and the pretreated NK cells were washed twice with PBS and then co-cultured with cell tracer violet-labeled K562 for 30 min. FasL expression of NK cells was examined by flow cytometry **(G)**. **(H)** Quantification of the percentage of FasL-expressing NK cells induced by K562 cells. Data were presented as Mean ± SD [*n* = 9–12 for **(C–F)**; *n* = 5 for **(H)**]. Samples were compared using one-way ANOVA with Dunnett's test. **P < 0.01, ***P < 0.001.

To investigate if NK cell metabolism also affects death receptor pathway-mediated killing, NK cells were pretreated with glucose-free medium or oligo plus R&A, and subsequently stimulated with anti-CD16 antibody and NKG2D ligand. The induction of FasL expression by anti-CD16 antibody and NKG2D ligand engagement was around 7% and was decreased by glucose starvation (Figures 7A,C,D). When stimulated with K562 cells, expanded NK cells showed a more drastic increase in FasL expression than the plate-bound anti-CD16 antibody and NKG2D ligand stimulated cells (Figures 7G,H). Similarly, the induction of FasL expression by K562 cells was significantly inhibited by glucose starvation. In contrast, the inhibition of OXPHOS exerted little effect on the NKR-induced FasL expression of NK cells (Figures 7B,E–H). Unstimulated *ex vivo* expanded NK cells constitutively express a substantial amount of TRAIL, and its expression could not be further increased by anti-CD16 and NKG2D ligand stimulation (Figure S2). Inhibition of glycolysis and OXPHOS had no impact on TRAIL expression.

Together, these results indicate that the cytotoxicity of expanded NK cells induced by NKR stimulation is mainly through CD107a expression, granzyme B secretion, and FasL expression, and are highly dependent on glycolysis.

## Discussion

Cellular metabolism plays a critical role in shaping immune responses by controlling immune cell differentiation and function. Resting immune cells (e.g., naïve and regulatory T cells) are relatively inert, so they depend primarily on OXPHOS to generate energy and to maintain their basal cell activities. However, pro-inflammatory and effector immune cells (e.g., cytotoxic T cell, Th1, and Th17 cells) have increased rates of glycolysis to generate ATP as well as intermediates for biosynthesis to support their functions (10). Recent studies found that resting NK cells have a low basal metabolic rate. However, under long-term cytokine stimulation, mouse or human NK cells upregulate glycolysis and OXPHOS, which are required for their effector functions (12, 13). In this current study, we investigated the role of metabolism in NKR-induced NK cell activation, which reflects the situation when NK cells are activated by target cells. We found that NKR stimulation elevates glycolysis and OXPHOS in human NK cells. We further demonstrated that IFN-γ production of NK cells induced by NKR stimulation requires both glycolysis and OXPHOS. More importantly, glycolysis is critical for supporting NK cell degranulation and FasL expression and their killing of target cells. Interestingly, inhibition of OXPHOS in the tumor microenvironment might be advantageous for the elimination of tumor cells by NK cells, because it does not affect NK cell cytotoxicity but renders tumor cells more susceptible to killing.

Cytokines and NKRs are two major pathways that regulate NK cell functions. NK cells distinguish self and non-self targets via their NKR expression on cell surface. CD16 acts as an Fc receptor that induces antibody-dependent cellular cytotoxicity. NKG2D is an important activating NKR which mainly recognizes stress-induced ULBP1-6 and MICA/B ligands on tumor or virally infected cells. Upon CD16 and NKG2D engagement, expanded and primary NK cells secrete cytokine IFN-γ (Figure 3) and induce cell cytotoxicity by boosting cell degranulation and FasL expression (Figures 2, 6, 7). Because *ex vivo* expanded NK cells, in our experiments, were cultured with IL-2 and feeder cells that express mbIL-15 and mbIL-21 for 14 days, they could be pre-activated and might have different metabolic state from the resting primary NK cells. Thus, we examined the metabolic changes in both expanded and primary NK cells activated by CD16 and NKG2D engagement to explore if they had distinct metabolic responses to NKR stimulation. Indeed, the expanded NK cells had low oxygen consumption rate for ATP production and showed limited increase in glycolytic rate when ATP synthesis was blocked in mitochondria, indicating that they probably depended more on glycolysis than OXPHOS in supplying ATP, while both glycolysis and OXPHOS were found to be important for ATP production in primary NK cells (Figures 1, 2). Interestingly, NKR stimulation induced similar metabolic reprogramming in both expanded and primary NK cells, i.e., both of them upregulated glycolysis and glycolytic capacity, and increased basal respiration, ATP-linked respiration, and maximal respiration in response to CD16 and NKG2D engagement (Figures 1, 2). However, ULBP1 apparently induced stronger glycolysis than MICA in expanded NK cells, although both ULBP1 and MICA are the ligands for NKG2D (Figures 1C,E). One possible reason is that the molar concentration of ULBP1 used in our experiments is higher than that of MICA, because we coated MICA and ULBP1 on plates with the same mass concentration, but the molecular weight of ULBP1 is smaller than MICA. Besides, the binding of MICA and ULBP1 to the plastic plate might be different, which could also affect the responses of NK cells to these ligands. Previous research reported that mouse NK cells activated by anti-NK1.1 and anti-Ly49D showed no increase in glycolysis and OXPHOS (11). Therefore, our results indicate that human and mouse NK cells might have different metabolic requirements upon NKR activation. Similar to our findings in NKR-activated NK cells, long-term stimulation of NK cells by cytokines (IL-2, IL-12, and IL-15) showed increased effector functions, which were accompanied by the upregulation of glycolysis and OXPHOS and the increase of nutrient transporters and mitochondrial mass (12, 13). Collectively, these results indicate both NKR and cytokine activation of human NK cells induce metabolic reprogramming in these cells.

We also investigated how metabolic reprogramming contributed to human NK cell effector functions, namely cytokine secretion and cytotoxicity. Previous studies showed that OXPHOS was required for cytokine-induced IFN-γ and granzyme B production by human primary NK cells, and glycolysis was required for the IFN-γ production by primary CD56^bright^ NK cells in response to IL-12 and IL-15 stimulation (13). Similarly, our data suggested that both expanded and primary NK cells require glycolysis and OXPHOS to support their production and secretion of IFN-γ upon their activation by NKR. Previous research found that the glycolytic enzyme GAPDH binds to the 3′ UTR of IFN-γ mRNA and inhibits the protein translation in T cells (20). Thus, it is possible that, when glycolysis increases, more GAPDH enzymes are released from IFN-γ mRNA and resulting in the increase in IFN-γ translation. Another possible mechanism by which glycolysis supports IFN-γ production might be through affecting histone acetylation of *Ifng*, as increased glycolysis produces more acetyl-CoA, which elevates histone acetylation of lysine 9 residue (H3K9Ac) at *Ifng* loci, thus increasing IFN-γ production (21).

Our data showed that glycolysis but not OXPHOS is critical for expanded NK cells to eliminate the target K562 cells. NK cells induced rapid clearance of K562 cells as they eliminated most of the target cells within 1.5 h at E:T ratio of 1:1. This rapid cytotoxicity is mainly through the granule exocytosis and FasL pathways. Recent studies emphasize the prerequisite of glycolysis in cytotoxicity and CD107a expression of educated NK cells (22, 23). Similarly, our data indicated that the NKR-activated NK cells also require glycolysis for NK cell cytotoxicity, mainly through supporting NK cell degranulation, granzyme B secretion, and FasL expression. It is possible that the rapid killing of target cells by NK cells requires fast metabolic mobilization, so NK cells are more reliant on glycolysis, which could be more rapidly activated than OXPHOS, for their cytotoxicity.

Interestingly, inhibiting OXPHOS in K562 cells made them more susceptible to NK cell killing but did not affect NK cell cytotoxicity. This is in line with a recent study showing that tyrosine kinase inhibitor-resistant chronic myeloid leukemia cells upregulate OXPHOS for their survival, and the combinational use of a tyrosine kinase inhibitor, imatinib with an OXPHOS inhibiting antibiotics, tigecycline can selectively kill chronic myeloid leukemia stem cells (24). Thus, the differentiated responses of NK and K562 cells toward OXPHOS inhibition make it a promising NK cell-based tumor treatment strategy in the clinic. Another study also reveals the ability of oligomycin to sensitize leukemia cells to tyrosine kinase inhibitor treatment. They demonstrated that the effect is partially through increasing the production of superoxide to induce cell apoptosis (25). Besides, the DNA damage caused by reactive oxygen species was shown to promote the expression of stress-induced activating ligands on multiple myeloma cells (26). Thus, it is possible that the inhibition of OXPHOS in K562 cells enhances their expression of activating ligands and elevates the superoxide production to increase K562 cells' susceptibility to NK cell killing.

In conclusion, our study reveals important metabolic requirements for NKR-activated human NK cells. Our data suggest that glycolysis and OXPHOS are both increased upon NKR activation of human NK cells. However, glycolysis is more important than OXPHOS for the effector functions of expanded NK cells. In addition, inhibition of OXPHOS in the tumor environment might be advantageous for NK cell-based immunotherapy.

## Data Availability Statement

The raw data supporting the conclusions of this article will be made available by the authors, without undue reservation, to any qualified researcher.

## Ethics Statement

The studies involving human participants were reviewed and approved by the Institutional Review Board of National University of Singapore (08-300). The patients/participants provided their written informed consent to participate in this study.

## Author Contributions

ZW, SX, and K-PL designed the work and wrote the manuscript. ZW and DG conducted the experiments. SW and LC provided critical reagents.

### Conflict of Interest

The authors declare that the research was conducted in the absence of any commercial or financial relationships that could be construed as a potential conflict of interest.
