# Supplementary material for: Glycolysis and Oxidative Phosphorylation Play Critical Roles in Natural Killer Cell Receptor-Mediated Natural Killer Cell Functions
Source: Front Immunol. 2020 Feb 20;11:202. doi: 10.3389/fimmu.2020.00202 (PMC7045049; doi:10.3389/fimmu.2020.00202)
Supplement: Supplementary file 1 [file Presentation_1.PPTX]

## Slide 1
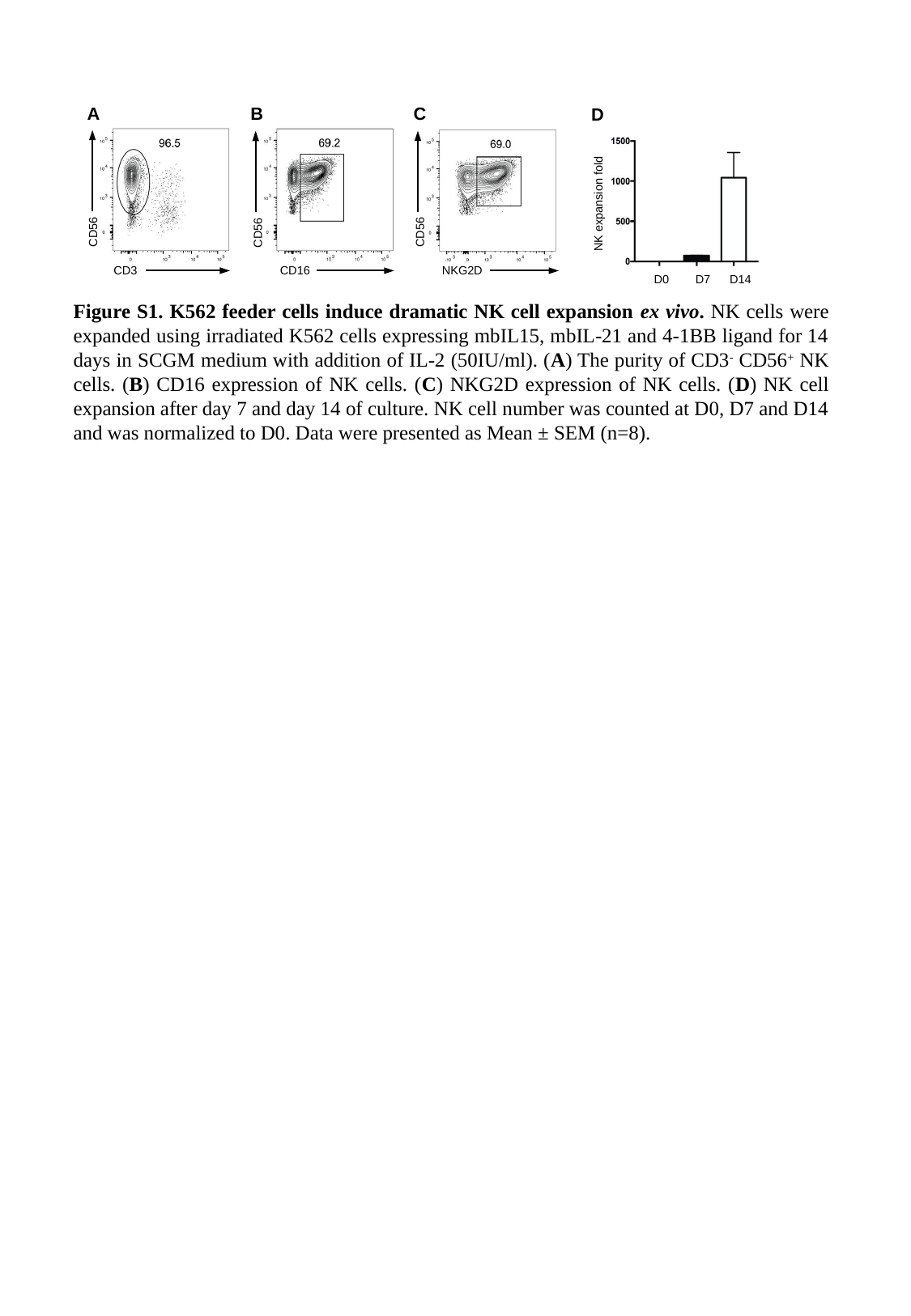

A B C
D
NK expansion fold
CD56
CD56
CD56
CD3
CD16
NKG2D
D0
D7 D14
Figure S1. K562 feeder cells induce dramatic NK cell expansion ex vivo. NK cells were expanded using irradiated K562 cells expressing mbIL15, mbIL-21 and 4-1BB ligand for 14 days in SCGM medium with addition of IL-2 (50IU/ml). (A) The purity of CD3- CD56+ NK cells. (B) CD16 expression of NK cells. (C) NKG2D expression of NK cells. (D) NK cell expansion after day 7 and day 14 of culture. NK cell number was counted at D0, D7 and D14 and was normalized to D0. Data were presented as Mean ± SEM (n=8).
