# Supplementary material for: Glycolysis and Oxidative Phosphorylation Play Critical Roles in Natural Killer Cell Receptor-Mediated Natural Killer Cell Functions
Source: Front Immunol. 2020 Feb 20;11:202. doi: 10.3389/fimmu.2020.00202 (PMC7045049; doi:10.3389/fimmu.2020.00202)
Supplement: Supplementary file 2 [file Presentation_2.PPTX]

## Slide 1
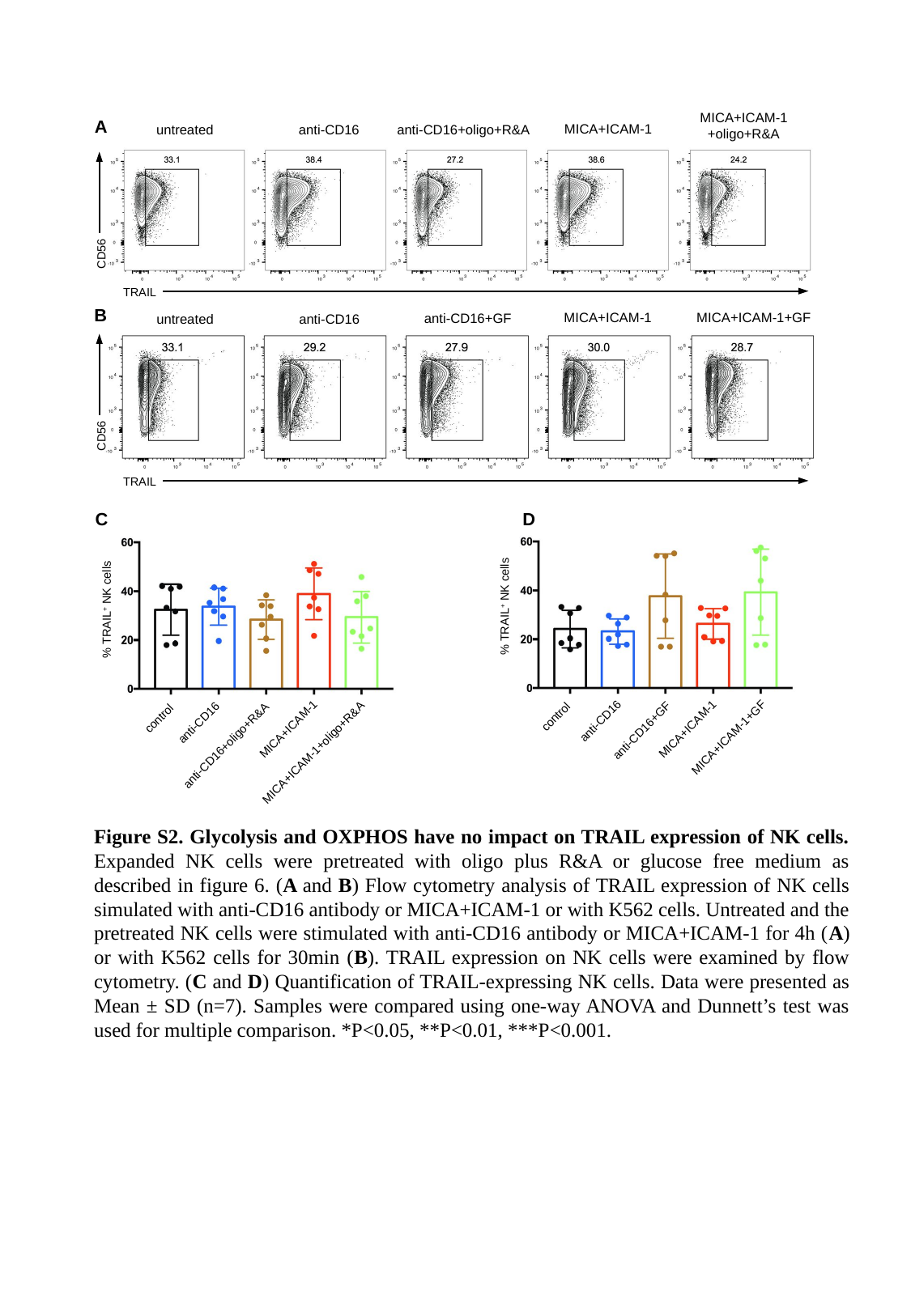

MICA+ICAM-1
+oligo+R&A
A
MICA+ICAM-1
anti-CD16+oligo+R&A
untreated
anti-CD16
CD56
TRAIL
B
MICA+ICAM-1
MICA+ICAM-1+GF
anti-CD16+GF
untreated
anti-CD16
CD56
TRAIL
C
D
% TRAIL+ NK cells
% TRAIL+ NK cells
control
control
anti-CD16
anti-CD16
MICA+ICAM-1
MICA+ICAM-1
anti-CD16+GF
MICA+ICAM-1+GF
anti-CD16+oligo+R&A
MICA+ICAM-1+oligo+R&A
Figure S2. Glycolysis and OXPHOS have no impact on TRAIL expression of NK cells. Expanded NK cells were pretreated with oligo plus R&A or glucose free medium as described in figure 6. (A and B) Flow cytometry analysis of TRAIL expression of NK cells simulated with anti-CD16 antibody or MICA+ICAM-1 or with K562 cells. Untreated and the pretreated NK cells were stimulated with anti-CD16 antibody or MICA+ICAM-1 for 4h (A) or with K562 cells for 30min (B). TRAIL expression on NK cells were examined by flow cytometry. (C and D) Quantification of TRAIL-expressing NK cells. Data were presented as Mean ± SD (n=7). Samples were compared using one-way ANOVA and Dunnett’s test was used for multiple comparison. *P<0.05, **P<0.01, ***P<0.001.
